# Control of arbuscular mycorrhiza development by nutrient signals

**DOI:** 10.3389/fpls.2014.00462

**Published:** 2014-09-11

**Authors:** Samy Carbonnel, Caroline Gutjahr

**Affiliations:** Faculty of Biology, Genetics, University of Munich (LMU)Martinsried, Germany

**Keywords:** arbuscular mycorrhiza, nutrient signaling, phosphate, MYCS element, P1BS element, symbiosis

Inorganic phosphate (P_i_), the main form of phosphorus used by plants, is one of the most important limiting factors for plant growth. In the soil soluble P_i_ that is readily available for uptake, occurs at very low concentrations (Schachtman et al., 1998). One adaptation of plants to low P_i_ availability is the symbiosis with arbuscular mycorrhiza fungi (AMF) of the phylum *Glomeromycota*. The fungi efficiently take up phosphate and other mineral nutrients and deliver them to the host, in exchange for carbohydrates. Thereby, arbuscular-mycorrhiza compatible plants have two P_i_ uptake pathways, which are defined by different sets of phosphate transporters: a direct uptake pathway through the epidermis and root hairs, and a symbiotic uptake pathway for the P_i_ provided by the fungus (Smith and Smith, 2011).

For successful symbiosis the fungus colonizes the root. This involves initial recognition *via* diffusible molecules, hyphal docking to the root surface by a hyphopodium, re-differentiation of plant cells and their subsequent penetration by fungal hyphae and formation of highly branched fungal arbuscules in the root cortex, which release mineral nutrients to the host (Gutjahr and Parniske, 2013). Plants control the degree of arbuscular mycorrhiza (AM) colonization depending on their nutritional status and it has been repeatedly reported that under high P_i_ supply, AM development is repressed (e.g., Menge et al., 1978; Braunberger et al., 1991; Balzergue et al., 2010; Breuillin et al., 2010). This suppressive effect of high P_i_ on root colonization by AMF is partially overruled by nitrogen (N) starvation, and to a lesser extent by potassium, calcium or iron starvation (Nouri et al., 2014), suggesting that plants control the symbiosis in function of their nutrient requirements according to Liebig's law of the minimum. The molecular mechanisms underlying the control of AM development by nutrient conditions are largely unknown. Conceptually, two scenarios are possible: AM development might be actively suppressed at high P_i_ conditions (Figures 1A,C). Alternatively or in addition, root cells might be conditioned by P_i_ starvation to actively promote AM formation (Figures 1B,D). At sufficient P_i_ supply this promotion might be simply absent. Here we examine the available literature for evidence for one or the other scenario.

**Figure 1:**
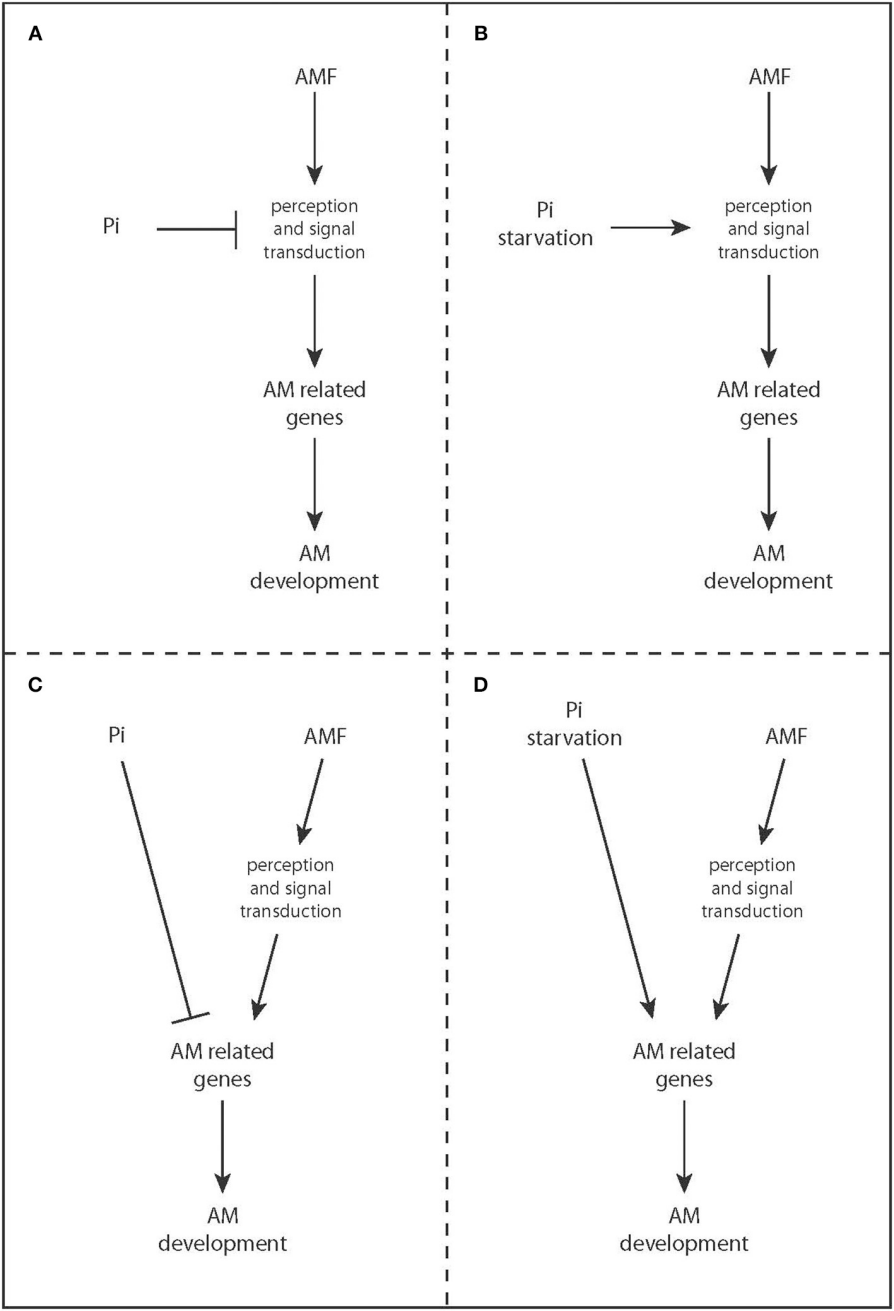
**Hypothetical models of how phosphate signaling might control AM development**. Other nutrients might influence AM development in a similar fashion as shown here for P_i_. **(A,B)** Perception of Myc factors, signal transduction through common SYM network or other symbiosis signaling pathways are directly targeted by P_i_ signaling. **(C,D)** P_i_ and symbiosis signaling co-regulate downstream genes that are required for AM development and maintenance (e.g., symbiotic phosphate transporters). **(A–C)** Signaling events that are generated in response to a high P_i_ status of the shoot actively inhibit AM development. **(B–D)** P_i_ starvation signaling is required to promote AM colonization.

Although several nutrients influence AM development (Nouri et al., 2014) most research has focused on the role of P_i_. Hyphopodium numbers on maize roots were inversely correlated with the P_i_ status of the shoot (Braunberger et al., 1991), indicating that AM suppression by high P_i_ occurs systemically. Indeed, split root experiments in pea and *Petunia*, showed an inhibition of AM colonization in the entire root system, even if only one half of the root system was fertilized with a high P_i_ concentration and the other half maintained a low P_i_ content (Balzergue et al., 2010; Breuillin et al., 2010). This calls for a long distance signal traveling from the shoot to the root to regulate AM colonization that might either suppress AM at high P_i_ or promote its development at low P_i_. Candidates for long-distance signaling molecules could be members of the miR399 family since they play a well-established role in systemic P_i_-starvation signaling (Lin et al., 2008; Pant et al., 2008; Gu et al., 2011). Interestingly, upon AM colonization, the expression of some miR399 family members was increased in *Medicago* and tomato leaves (Branscheid et al., 2010; Gu et al., 2014). Consistently, transcript levels of the miR399 target, PHO2 an ubiquitin E2 conjugase, that mediates the degradation of proteins required for phosphate starvation responses (Liu et al., 2012; Park et al., 2014), remained low (Branscheid et al., 2010). It was postulated that increased expression of miR399 family members might serve to keep phosphate starvation responses high to allow continuous colonization, in spite of increased shoot phosphate content resulting from functional symbiosis (Branscheid et al., 2010). However, miR399 over-expression failed to support colonization under high P_i_ supply (Branscheid et al., 2010), indicating that other regulatory mechanisms link AM development to the nutrient status of the plant.

Plant endosymbiosis (AM and root nodule symbiosis) development requires a common set of genes called common SYM genes. Their protein products belong to a signal transduction cascade that is triggered by perception of fungal signals (Myc factors) through receptor-like-kinases (Gough and Cullimore, 2011). Myc factor perception induces nuclear Ca^2+^-spiking that is decoded by a nuclear localized calcium-calmodulin kinase (CCaMK) and leads to transcriptional activation of symbiosis-related genes by the transcription factor CYCLOPS (Genre et al., 2013; Singh et al., 2014). One potential explanation for suppression of AM development at high P_i_ could be the repression of common SYM signaling (Figure 1A). In *Petunia*, the expression of the putative ortholog of NOD FACTOR RECEPTOR 5, which is required for Nod factor perception in legumes, is decreased at high P_i_ (Breuillin et al., 2010). Although *Petunia* does not form root nodule symbioses this receptor might be involved in perception of fungal signaling molecules and reduced receptor availability might lead to abortion of colonization (Figure 1A, Breuillin et al., 2010). However, the expression of downstream common SYM genes involved in the generation and interpretation of Ca^2+^-spiking is not affected (Breuillin et al., 2010) and high P_i_ did not reduce the ability of *Medicago* rhizodermis cells to trigger nuclear Ca^2+^-spiking in response to rare hyphopodia or germinating spore exudates (Balzergue et al., 2013). Therefore, it is unlikely that receptor availability at the rhizodermis limits colonization. However, it remains open whether it affects colonization in the cortex (Breuillin et al., 2010; Op Den Camp et al., 2011). Alternatively, P_i_ might alter the signal transduction cascade either downstream of calcium spiking or influence a pathway that operates in parallel with common SYM signaling (Figure 1).

Good candidates for such downstream or parallel mechanisms are phytohormone signaling modules, as they steer developmental responses to the nutrient environment (Rubio et al., 2009) and regulate AM formation (Foo et al., 2013; Bucher et al., 2014; Gutjahr, 2014). For example strigolactones are exuded into the rhizosphere and stimulate germination, hyphal branching and metabolic activity of AM fungi (Akiyama et al., 2005; Besserer et al., 2006, 2008), which increases AM colonization (Gomez-Roldan et al., 2008; Foo et al., 2012; Gutjahr et al., 2012; Kohlen et al., 2012; Kretzschmar et al., 2012; Yoshida et al., 2012). At high P_i_ conditions the number of transcripts encoding strigolactone biosynthesis enzymes as well as strigolactone exudation from roots are reduced (Yoneyama et al., 2007; López-Ráez et al., 2008; Balzergue et al., 2010, 2013; Breuillin et al., 2010). However, exogenous supply of the synthetic strigolactone GR24 could not restore colonization at high P_i_ availability (Balzergue et al., 2010; Breuillin et al., 2010), indicating that the reduced strigolactone exudation is not the main reason for low AM colonization. Gibberellins (GAs) negatively regulate AM development. In *Medicago* and pea exogenous GA treatment of roots blocked arbuscule formation but did not affect the colonization with intraradical hyphae (Floss et al., 2013; Foo et al., 2013), while in rice it generally reduced intraradical colonization (Yu et al., 2014). Consistently, DELLA proteins, which are repressors of GA signaling are required for AM development (Floss et al., 2013; Foo et al., 2013; Yu et al., 2014). *Arabidopsis* plants grown at P_i_ starvation conditions accumulate DELLA proteins and reduced levels of GA (Jiang et al., 2007). Thus, the GA signaling module has the potential to regulate AM development and in particular arbuscule formation according to the plant phosphate status (Floss et al., 2013). Interestingly, arbuscule formation of the *L. japonicus* common SYM mutant *cyclops* can be restored by overexpression of a resistant DELLA version. The DELLA/GA module is therefore a good candidate regulator of arbuscule development either downstream of or in parallel with common SYM signaling (Floss et al., 2013; Gutjahr, 2014). However, it remains to be tested whether transgenic expression of a resistant DELLA can counteract the negative impact of high P_i_ supply on AM symbiosis.

Symbiotic P_i_ uptake occurs in cortex cells that are colonized by arbuscules (Javot et al., 2007a). Arbuscules are surrounded by a plant derived periarbuscular membrane that hosts a specific set of membrane proteins (Pumplin and Harrison, 2009). Importantly, it contains symbiotic phosphate transporters (PT4/PT11), which import phosphate ions that are released by the arbuscule into the plant cell (Javot et al., 2007b; Yang et al., 2012). *Medicago pt4* and rice *pt11* mutants revealed that PT4/PT11 is not only essential for AM-mediated phosphate uptake but also for arbuscule maintenance (Javot et al., 2007b; Yang et al., 2012) demonstrating that P_i_ import is crucial for wild-type arbuscule dynamics. It has been suggested that the P_i_ ion itself could act as a local, cell-autonomous signal that triggers accommodation and maintenance of the arbuscule by the host cell (Javot et al., 2007a; Yang and Paszkowski, 2011). This notion is supported by the rice *pt13* mutant, which is deficient in a second AM-induced phosphate transporter called PT13. It is not impaired in symbiotic phosphate uptake, but in arbuscule maintenance. Thus, OsPT13 might act as a P_i_ sensor rather than a transporter (Yang et al., 2012). Arbuscule lifespan in roots of the *Medicago pt4* mutant is restored by growing the plant at low nitrogen (N) concentrations (Javot et al., 2011). This indicates that not only P_i_- but also N-delivery can support arbuscule maintenance in a cell-autonomous fashion.

Analysis of the promoter regions of symbiotic PTs in different species revealed two conserved cis-elements called MYCS (or CTTC) and P1BS that are often located close to each other (Karandashov et al., 2004; Chen et al., 2011). Deletion of each of these elements from PT promoters driving a GUS reporter gene showed that they are both essential for colonization-responsive promoter activation (Chen et al., 2011), suggesting that at least 2 transcriptions factors (TFs) co-regulate the expression of symbiotic PTs. The MYCS element is over-represented in mycorrhiza-regulated genes and four repeats of the MYCS element alone are sufficient to drive GUS-expression in colonized areas of the root (Lota et al., 2013). Thus, the P1BS element is dispensable when MYCS is taken out of context. The P1BS motif is common to many promoters of P_i_ starvation-induced genes and is targeted by central regulators of P_i_ starvation responses, the MYB transcription factor PHR1 and its homologs (Bustos et al., 2010). Thus, promoter induction of symbiotic phosphate transporters and other mycorrhiza-responsive genes, likely requires simultaneous activation by symbiosis signaling and P_i_ starvation signaling (Figure 1D). Repressed expression of a transgene containing 4xMYCS-GUS after fertilization of colonized transgenic roots of *Lotus japonicus* with high P_i_ for 2 weeks seems to contradict this hypothesis (Lota et al., 2013). However, in *Petunia* it has been shown earlier that such a long period of phosphate replenishment leads to decreased root colonization while expression of a symbiotic PT gene is already suppressed after 2–4 days of high P_i_ supply (Breuillin et al., 2010). Therefore, 2 weeks after P_i_ replenishment, MYCS activation is probably indirectly affected due to fungal senescence and cessation of symbiotic signaling. Nevertheless, the important role of symbiotic PTs in AM symbiosis maintenance (Javot et al., 2007b; Yang et al., 2012) and their transcriptional regulation by P_i_ conditions (Nagy et al., 2009; Breuillin et al., 2010) makes them possible targets of AM developmental control by nutrients. Taking together this assumption with the phenotype of *pt4*/*pt11* and *pt13* mutants creates an important paradox: on one hand systemic P_i_ starvation is required to allow the expression of symbiotic PTs; on the other hand symbiotic PTs themselves need to deliver the phosphate that will allow arbuscule formation and maintenance (Javot et al., 2007b; Breuillin et al., 2010; Yang et al., 2012). How plants integrate these opposing situations—i.e., simultaneous requirement of systemic P_i_ starvation and of cell-autonomous symbiotic P_i_ delivery—represents a very intriguing question for future research.

In summary, the mechanisms of how AM development is controlled by nutrient signaling are yet elusive. However, circumstantial evidence suggests that control occurs at multiple levels, and includes nutrient, phytohormone and symbiosis as well as systemic and cell-autonomous signaling. The signal operating in systemic shoot-to-root P_i_ signaling remains to be found. Strigolactone exudation, the DELLA/GA signaling module and the P1BS element-binding transcription factor are excellent candidates for local mediators between nutrient status and AM development in the root. Additionally, the co-occurrence of MYCS and P1BS cis-elements in AM-inducible promoters strongly suggests that P_i_ starvation signaling and AM signaling are required simultaneously for symbiotic gene expression and consequently colonization (Figure 1D). Transgenic manipulation using dominant negative and dominant active candidate signaling components with the aim to restore AM development at sufficient P_i_ should help to pinpoint the important regulators and to detangle how plants integrate symbiosis, phytohormone and nutrient signaling to control AM development in function of their nutrient status.

## Conflict of interest statement

The authors declare that the research was conducted in the absence of any commercial or financial relationships that could be construed as a potential conflict of interest.
